# KM110329 in adult patients with atopic dermatitis: a randomised, double-blind, placebo-controlled, multicentre trial – study protocol

**DOI:** 10.1186/1472-6882-13-335

**Published:** 2013-11-27

**Authors:** Chunhoo Cheon, Sunju Park, Jeong-Su Park, So-Mi Oh, Soobin Jang, Ho-Yeon Go, Bo-Hyoung Jang, Yong-Cheol Shin, Seong-Gyu Ko

**Affiliations:** 1 Center for Clinical Research and Drug Development, College of Korean Medicine, Kyung Hee University, Hoegi-dong, Seoul, Republic of Korea; 2 Department of Preventive Medicine, College of Korean Medicine, Kyung Hee University, Seoul, Republic of Korea; 3 Department of Preventive Medicine, College of Korean Medicine, Daejeon University, 62 Daehak-ro, Dong-gu, Daejeon 300-716, Republic of Korea; 4 Department of Internal Medicine, College of Korean Medicine, Semyung University, Bongbang-dong, Chungju 380-960, Republic of Korea

**Keywords:** Atopic dermatitis, Functional food, Natural product, Herbal medicine

## Abstract

**Background:**

Atopic dermatitis is a chronic inflammatory skin disease with a high prevalence rate and a large socioeconomic cost. There are many treatments for atopic dermatitis, such as antihistamine, tacrolimus and corticosteroids. However, due to concern about the adverse effects, many patients seek alternative treatments. In this context, natural products are gaining interest. KM110329 is a functional food consisting of four herbs that are beneficial to skin health. The purpose of this study is to assess the efficacy and safety of KM110329 for atopic dermatitis.

**Methods/design:**

This study is a randomised, double-blind, placebo-controlled and multicentre trial of KM110329. For this study, we will recruit 66 atopic dermatitis patients of both sexes, with ages ranging from 18 to 65, from three university hospitals. The participants will receive either KM110329 or a placebo twice a day for 8 weeks. The primary end point will be a change in the scoring atopic dermatitis (SCORAD) index. The secondary end points will include changes to the dermatology life quality index (DLQI) and transepidermal water loss (TEWL), among others. The outcomes will be measured at every visit. The study will be continued for 8 weeks and will include five visits with each subject (at screening and at 0, 1, 4 and 8 weeks).

**Discussion:**

This trial will provide research methodologies for evaluate clinical efficacy and safety of KM110329 in adult patients with atopic dermatitis. In addition, we will evaluate the changes in the general skin health status and quality of life.

**Trial registrations:**

ClinicalTrials.gov NCT01692093.

## Background

Atopic Dermatitis (AD) is a chronic inflammatory pruritic skin disease that is often associated with other atopic disorders, such as allergic rhinitis and asthma [1]. AD is characterised by poorly defined erythema with oedema, vesiculation, weeping in the acute stage, and skin thickening (lichenification) in the chronic stage [2].

The prevalence of AD has risen substantially in many countries in recent decades. AD not only leads to a low quality of life, but it also causes depression. AD affects the patients as well as their parents and other family members as well [3]. Therefore, the socioeconomic cost of AD is increasing. In Korea, the total number of people who visit the hospital for AD is 1,035,680 (57^th^ of total 500 disease), and the total was 62,381,288,330 Won in 2010. In addition to the medical market for AD treatments, there is also an enormous cosmetic market totalling approximately 100 billion Won.

The existing treatments for treat AD include topical corticosteroids, emollients, topical tacrolimus, topical 1% pimecrolimus, oral antihistamines, refined-coal tar, topical doxepin and oral corticosteroids. However, these treatments can cause adverse reactions, such as stinging, burning, drowsiness, itching, dyspepsia and hypertension.

Therefore, a demand for novel treatments exists, and functional foods made from natural products have received considerable attention due to the perception that they have no adverse effects [2].

KM110329 is compound found in Rubi Fructus, Houttuyniae Herba, Rehmanniae Radix, and Betulae Platyphyllae Cortex.

According to herbology textbooks and the World Health Organization (WHO) glossary, the Korean medicinal efficacies of each component of KM110329 are as follows [4,5].

The effects of Rubi Fructus are to tonify the kidney, to secure its essence and to reduce urination. Rubi Fructus works as a treatment for insufficient consolidation due to kidney deficiency, and it also has beneficial effects on the skin.

Houttuyniae Herba has the function of clearing away heat, detoxifying the body, dissolving boils and draining pus. As a home remedy, it is used to treat furuncle.

Rehmanniae Radix clears heat, engenders fluid, cools the blood to stop bleeding and treats agitation, thirst and skin irritation caused by heat.

Betulae Platyphyllae Cortex mitigates pruritus and skin pain by clearing heat, draining dampness, dispelling phlegm and suppressing cough, dispersing swelling, and detoxifying the body.

Some studies have reported the components of KM110329 have effect on atopic dermatitis. Recent studies suggests that Rubi fructus regulates anaphylaxis and have anti-inflammatory effect [6,7]. Rehmanniae Radix prevents skin allergic reaction and treats allergen-induced atopic dermatitis in mice by suppressing the expression of cytokines, chemokines and adhesion molecules [8,9]. Houttuyniae Herba suppresses Th2 immune response and regulates inflammatory process [10,11]. Betulae Platyphyllae Cortex inhibits the development of atopic dermatitis-like skin lesions in NC/Nga mice through the suppression of the Th2 cell response. It also attenuates mast cell-mediated allergic inflammation in vivo and in vitro [12,13].

The aim of the present study is to determine the clinical efficacy and safety of KM110329 for AD.

We will conduct a randomised, double-blind, placebo-controlled, multicentre trial of KM110329 in adult patients with AD. Because of the absence of a gold standard for the treatment of AD, this trial will be conducted to determine the effectiveness of this functional food in comparison with a placebo-controlled group.

## Methods/design

**Figure 1:**
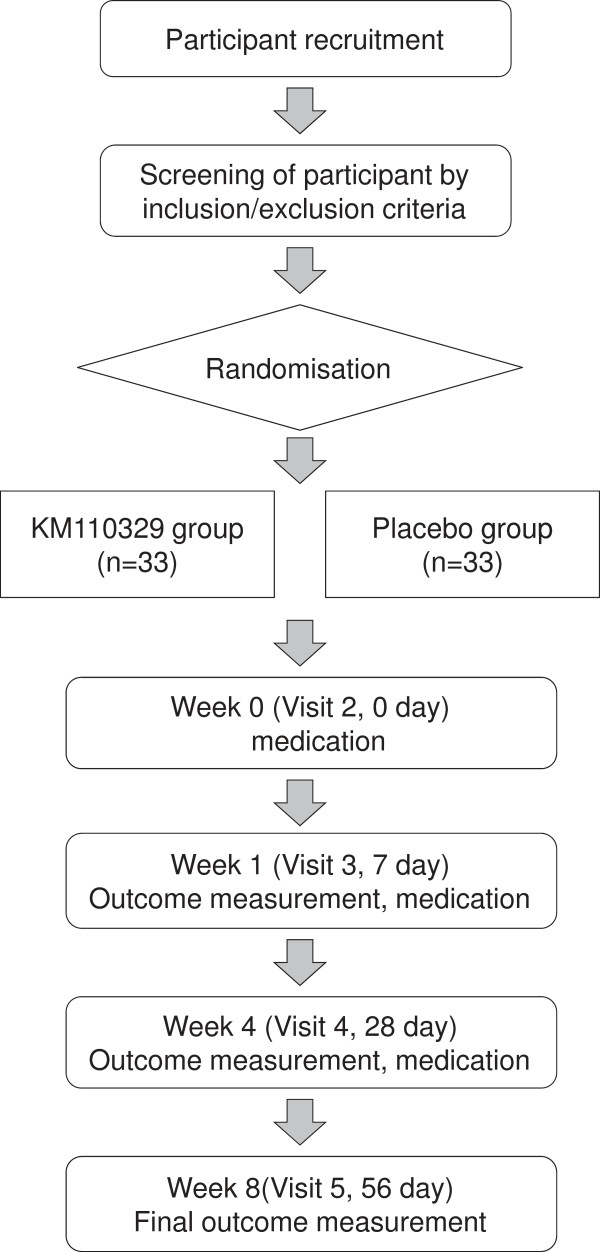
Study flow chart.

A randomised, double blind, placebo-controlled, multicentre trial will be conducted at the Seoul St. Mary’s Hospital, Chung-ang University Hospital, and Kyung Hee University Hospital in Gangdong. Participants fulfilling the eligibility criteria will be selected. The enrolled participants will be randomly allocated to two parallel groups corresponding to the KM110329 and placebo arms. Each participant will be examined for signs and symptoms of AD before and after taking the functional food. Figure 1 shows the schematic flow of the study.

### Recruitment

Subjects will be recruited through three routes. Patients who visit the trial hospitals and meet the criteria will be recommended by the physician in charge. Patients who see the trial poster on bulletin boards or advertisements in newspapers will visit the trial site voluntarily.

### Inclusion criteria and exclusion criteria

The inclusion and exclusion criteria are shown in Inclusion and exclusion criteria section.

### Inclusion and exclusion criteria

Inclusion criteria

1. Men and women ages 18 to 65 years

2. Individuals diagnosed with atopic dermatitis according to the criteria of Hanifin and Rajka

3. Individuals with mild to moderate atopic dermatitis (objective SCORAD ≤ 40)

4. Written informed consent for participation in the trial

Exclusion Criteria

1. Severe skin disease other than atopic dermatitis

2. Secondary infection with bacteria, fungi, or virus

3. Uncontrolled hypertension (SBP > 145 mmHg or DBP > 95 mmHg)

4. Severe liver disability (2.5-fold the normal high range value for ALT, AST)

5. Severe renal disability (sCr > 2.0 mg/dl)

6. Women who are pregnant, lactating, planning a pregnancy or women of child-bearing age who do not agree to use proper contraception

7. Use of oral steroids, oral antibiotic or other immunosuppressants within the past 4 weeks

8. Treated by systemic photochemotherapy within the past 4 weeks

9. History of drug abuse

10. Hypersensitivity to Rubi Fructus, Houttuyniae Herba, Rehmanniae Radix, or Betulae Platyphyllae Cortex

11. Use of other investigational products within the past two months

### Subject withdrawal criteria

The subjects who meet the criteria listed in Subject withdrawal criteria section will be discontinued from treatment. The participants who are withdrawn after randomisation will be followed for outcomes.

### Subject withdrawal criteria

1. Use of any forbidden medication or treatment during the trial that could affect the study result

2. Subject’s withdrawal of consent

3. Occurrence of a serious adverse event

4. Detection of eligibility violations, occurrence of other significant protocol violations during the trial

5. Investigator’s decision to terminate the process for the sake of the subject’s health

### Sample size

Sample size calculations were performed to determine the number of participants needed to detect the effect sizes. We obtained an effect size from the previous study and based on the agreement of experts [14]. The results from the previous study were used to calculate the standard deviation [15]. The mean difference and standard deviation of the scoring atopic dermatitis (SCORAD) index were 10 and 13.576, respectively. The following formula was used to estimate the sample size.

$$\begin{array}{ll} n & =\frac{2{\left(z_{\alpha /2}+z_{\beta }\right)}^{2}\sigma^{2}}{{\left(\mu_{c}-\mu_{t}\right)}^{2}}\\ & =\frac{2\times {\left(1.96+0.84\right)}^{2}\times 13.576^{2}}{10^{2}}\\ & =28.932 \end{array}$$

The calculation was performed using 80% power, a 5% significance level, and a 10% dropout rate. The required sample size is approximately 33 participants for each group. Therefore, a total of 66 participants are needed for this trial.

### Randomisation and allocation

The study subjects who satisfy the eligibility criteria will be randomised using a computer program at an independent centre (Kyung Hee University Center for Clinical Research and Drug Development; KCRD). Block randomisation with block size of 4 will be performed. The study subjects will be assigned to one of two groups with a 1:1 allocation ratio by investigator in each study site. Random numbers will be sent to each centre, and a randomisation table will be maintained by KCRD during the research period. Opaque sealed envelopes containing serial numbers will be delivered to each centre. The randomisation table kept in an opaque sealed envelope by the contract research organisation (CRO; KCRD) should be opened according to Standard Operating Procedures (SOPs).

### Blinding

Both the investigator and the subject will be blinded regarding the assignment of the study drugs. The CRO of the sponsor will label the investigational drugs using the randomisation code number. The labelled experimental products will be provided to the trial sites by the CRO.

### Treatment protocol

The participants will receive KM110329 or a placebo drug for eight weeks. They will take 2 tablets by mouth with water twice a day after meals.

### Interventions

Hanpoong Pharm and Foods Co., Ltd. produced the KM110329 and the placebo according to Korea Good Manufacturing Practice (KGMP) standards. KM110329 is a brown-coloured, round-shaped tablet. It is compound of Rubi Fructus, Houttuyniae Herba, Rehmanniae Radix, and Betulae Platyphyllae Cortex. The placebo medicine is made of lactose, corn starch and food colouring, and it has a similar appearance, shape, weight, taste and colour as KM110329.

### Primary outcome measurement

The primary outcome in the present study is the change in the SCORAD index between the baseline (Visit 2) and after the treatment (Visit 5). The SCORAD index is a clinical tool for assessing the severity of atopic dermatitis as objectively as possible. The SCORAD index will be measured at every visit.

### Secondary outcome measurement

Secondary outcome measurements include the change in the Dermatology Life Quality Index (DLQI), the total IgE level, eosinophil counts, IL-4, IL-5, IL-13, TEWL (Transepidermal Water Loss), hydration in the stratum corneum, and KiFDA-HM-AD. The study schedule is detailed in Table 1.

**Table 1:** Study schedule of KM110329 clinical trial (8 weeks)

	**Screening**	**Treatment period**			
	**Visit 1**	**Visit 2**	**Visit 3**	**Visit 4**	**Visit 5**
	**(D − 7)**	**(D 0)**	**(D 7)**	**(D 28)**	**(D 56)**
**Informed consent**	**√**				
**Demographic characteristic eristic**	**√**				
**SCORAD index**	**√**	**√**	**√**	**√**	**√**
**Dermatology Life Quality Index**		**√**	**√**	**√**	**√**
**KiFDA-HM-AD**		**√**	**√**	**√**	**√**
**Laboratory tests**^**1**^	**√**	**√**			**√**
**Test for chronic hepatitis**^**2**^	**√**				
**Drug use history**	**√**				
**Concomitant medication**	**√**	**√**	**√**	**√**	**√**
**Pregnancy test**	**√**	**√**	**√**	**√**	**√**
**Medical/drug use history**	**√**	**√**	**√**	**√**	**√**
**Adverse event**			**√**	**√**	**√**
**Compliance calculation**			**√**	**√**	**√**
**Inclusion/exclusion criteria check**	**√**				
**Randomisation**		**√**			

^1^CBC (RBC, Hb, Hct, WBC, Platelet, ESR), Immunological tests (Eosinophil, IgE, IL-4, IL-5, IL-13), Liver function tests (AST, ALT), Renal function tests (BUN, Creatinine).

^2^Anti-HBs, HBsAg.

### Safety outcomes

All variables related to the safety assessment, such as vital signs, physical examinations, laboratory tests (complete blood cell count, liver function test, renal function test) and adverse events (AEs) will be documented on the case report form (CRF) at every visit.

### Statistical analysis

#### Efficacy assessment

Statistical analyses will be performed for both the ITT (intention-to treat, all randomly assigned participants) and PP (per-protocol, participants completed the trial without any protocol deviations) data sets. The missing values will be imputed by the last observation carried forward (LOCF) method. The continuous variables will be displayed as the mean ± SD(standard deviation), and the categorical variables will be displayed as the n (%). The baseline characteristics will be compared by either a Student’s *t*-test for continuous variables or the χ^2^ test (Fisher’s exact test will be used when the expected value is < 5) for the categorical data. Alternatively, McNemar’s test will be used if the normality assumption is not satisfied for the continuous variables. For the primary outcome measures, the mean differences from the baseline values to the end of treatment will be compared using an independent T-test.

#### Safety assessment

All participants will report any adverse events that they experience while undergoing the intervention at every visit. Every adverse event will be described in the CRF. If the adverse event is severe and associated with the trial, the participant will be withdrawn from the trial, and the appropriate treatment will be given to him or her. For the safety assessment, a liver function test, blood cell count test and physical examination will be performed at Visits 1, 2 and 5. The safety-related variables will be analysed using the ITT method.

### Data and safety monitoring

To maintain the quality of this trial, monitoring will be conducted by Kyung Hee University Center for Clinical Research and Drug Development (KCRD; the CRO). Every institution participating in the trial will be monitored while this trial is in progress using standard operating procedures.

### Ethics

The present study has been approved by the institutional review boards (IRBs) at each of the three institutions, which are the Catholic University of Korea Seoul St Mary’s Hospital (reference KC12HNME0047), the Chung-ang University Hospital (reference C2011197), and the Kyung Hee University Hospital at Gangdong (reference KHNMC-OH-IRB 2011–019). Written informed consent will be obtained from each participant prior to enrolment. The trial will be performed in compliance with the Helsinki Declaration and according to Good Clinical Practice as described by the Korea Food & Drug Administration.

## Discussion

The present study seeks to investigate the clinical efficacy and safety of KM110329 for AD.

The desire for well-being and to improve one’s quality of life motivates consumers to purchase functional foods, thus opening an enormous new market.

The functional food market in Korea was introduced in the 1990s. It underwent the early stages of growth from 2002 to 2005, and it reached maturity in late 2006, valued at more than 1,280 billion Won. In the USA, the functional food market grows more than 6% annually, and it was estimated to reach 22.5 billion dollars in 2006 [16].

Despite the recent demand for functional food, there are only a few trial protocols to determine the clinical efficacy and safety of functional foods. In the present study, we will evaluate the efficacy of KM110329, a functional food consisting of four herbs that have been shown to be beneficial for skin health.

A study protocol for the treatment of AD using the herbal medicine Hwangryunhaedoktang has already been published [17], however, the present study has several distinctive features. First, this will be the first trial to investigate the efficacy of the functional food KM110329 for the treatment of AD. In the present study, we will assess not only the usual outcome measurements, such as SCORAD, DLQI, and IgE, but also TEWL and hydration in the stratum corneum to assess the overall health status of the skin.

Second, to the best of our knowledge, this will be the first trial protocol using KiFDA-HM-AD to evaluate severity of AD. KiFDA-HM-AD is an evaluation endpoint for clinical trials of herbal medicinal products related to AD [18]. The Korea Food and Drug Administration (KFDA) developed and deployed this assessment tool. This trial may provide a basic assessment of the reliability and validity of KiFDA-HM-AD by comparing the outcomes of the KiFDA-HM-AD with those of the SCORAD and DLQI.

We hope that this trial protocol will be a reference for designing further clinical trials for functional foods to improve skin health.

## Abbreviations

SCORAD index: Scoring atopic dermatitis index; DLQI: Dermatology life quality index; TEWL: Transepidermal water loss; AD: Atopic dermatitis; KCRD: Kyung Hee University Center for Clinical Research and Drug Development; SOPs: Standard operating procedures; CRO: Contract research organisation; KGMP: Korea good manufacturing practice; AEs: Adverse events; CRF: Case report form; ITT: Intention-to treat; PP: Per-protocol; LOCF: Last observation carried forward; IRBs: Institutional review boards.

## Competing interests

The authors declare that they have no competing interests.

## Authors’ contributions

CHC, JSP, SJP, SMO, SBJ, HYG and BHJ have written the initial manuscript for this trial and calculated the sample size, and they will monitor this trial. YCS and SGK have edited the first manuscript. SGK has conducted all the procedures for this protocol. All authors have read and approved the final manuscript.

## Pre-publication history

The pre-publication history for this paper can be accessed here:

http://www.biomedcentral.com/1472-6882/13/335/prepub
